# Haematological and serum biochemical values in Norwegian sled dogs before and after competing in a 600 km race

**DOI:** 10.1186/s13028-019-0453-5

**Published:** 2019-04-25

**Authors:** Tuva Holt Jahr, Marte Ekeland Fergestad, Ola Brynildsrud, Hege Brun-Hansen, Ellen Skancke

**Affiliations:** 1 Department of Companion Animal Clinical Sciences, Faculty of Veterinary Medicine, Norwegian University of Life Sciences, PO Box 8146 Dep, 0033 Oslo, Norway; 2 Department of Food Safety and Infection Biology, Faculty of Veterinary Medicine, Norwegian University of Life Sciences, PO Box 8146 Dep, 0033 Oslo, Norway; 3 Department of Infectious Disease Epidemiology and Modelling, Norwegian Institute of Public Health, Lovisenberggata 8, 0456 Oslo, Norway

**Keywords:** Biochemistry, Haematology, Long distance race, Muscle enzymes, Sled dog

## Abstract

**Background:**

Long-distance racing is known to cause alterations in haematological and serum biochemical parameters in sled dogs. Given that finishing status reflects the physical condition in dogs completing a race, such variations will mainly be the result of physiological adaption achieved during endurance exercise. However, changes observed in withdrawn dogs may indicate pathological conditions. The aim of this study was to reveal changes in haematological and serum biochemical values in sled dogs participating in a long-distance race, with emphasis on the withdrawn dogs. Sixty-five sled dogs participated in a clinical prospective cohort study: 46 dogs competed in the 600 km race (25 finishing and 21 withdrawn dogs), and 19 dogs served as controls. Blood sampling was performed early in the training season and after the race.

**Results:**

When compared to control dogs, both withdrawn and finishing dogs showed significant increases in neutrophil count, C-reactive protein, blood urea nitrogen and sodium/potassium ratio. Significant decreases were found in erythrocytes and eosinophil cell count, and in haematocrit, haemoglobin, total protein, albumin, globulin, creatinine, potassium and calcium levels. Finishing dogs presented significant increases in white blood cells, large unstained cells, monocyte count and cortisol level compared to control dogs. In contrast, withdrawn dogs had significant elevations in alanine aminotransferase and alkaline phosphatase activity, as well as parameters associated with muscle metabolism, such as aspartate aminotransferase, creatine kinase and phosphorus concentration.

**Conclusions:**

Competing sled dogs experienced minor changes in blood parameters in general, mainly revealing the same pattern among withdrawals and finishers. This might indicate that numerous changes simply reflect physiological adaption due to endurance exercise. However, the serum concentration of muscle enzymes was significantly increased only in the withdrawals, and were well above reference ranges. This reflects muscle degradation, which could be the main cause of performance failure in some of the withdrawals.

**Electronic supplementary material:**

The online version of this article (10.1186/s13028-019-0453-5) contains supplementary material, which is available to authorized users.

## Background

Endurance exercise in sled dogs is associated with haematological and serum biochemical changes [1–4], although the underlying causes are not completely known. Most changes are considered to reflect normal physiological alterations due to heavy exercise, extreme energy demands and environmental challenges during racing. However, many dogs are withdrawn due to physical exhaustion, lameness or medical reasons, including pneumonia, blood loss secondary to gastric ulceration, enteritis and rhabdomyolysis [5, 6]. Hence, some of the observed changes during racing may have pathophysiological origin. In addition, factors such as breed, sex and age, as well as different handling and feeding strategies prior to and during racing, may have an influence.

The aim of this study was to identify and evaluate serum biochemical and haematological alterations in dogs participating in a genuine endurance race, focusing on the withdrawn sled dogs. The results from blood samples retrieved early in the training season were compared with samples taken immediately after race participation. Dogs training for the race, but not competing, served as the control group. The results from withdrawn and finishing dogs were compared to the controls.

## Methods

### Study design and population

#### Clinical prospective cohort study

Sled dogs (n = 65) from four different mushers were included. They were all possible candidates for participation in “Femundløpet”, a Norwegian long-distance race of 600 km, in February 2013. All dogs participated in training during the fall and winter 2012–2013. The dogs were classified by age (years), sex (female/male) and ownership (owner 1–4). Information regarding health, training and feeding routines during the training season was collected from the mushers via questionnaires.

The dogs were divided into three cohorts based on participation and outcome of the race: finishing dogs (cohort 1) (n = 25), withdrawn dogs (cohort 2) (n = 21) and non-participation/control dogs (cohort 3) (n = 19) (Table 1). Cohort 2 was defined as dogs that discontinued the race. The reasons for withdrawal were not specified. The study group has been featured in a previous report [7].

**Table 1:** Cohorts and sampling groups. Blood samples from each dog were retrieved prior to the training season (A sample), and after the race (B sample). The dogs were divided in three groups; finishers, withdrawals and non-participants/controls

Blood sampling during training season, fall 2012 (A samples)		
Cohort 1 (A1 samples)	Cohort 2 (A2 samples)	Cohort 3 (A3 samples)
In training	In training	In training
n = 25	n = 21	n = 19

Blood sampling after the race, 2013 (B samples)		
Cohort 1 (B1 samples)	Cohort 2 (B2 samples)	Cohort 3 (B3 samples)
Finishing dogs	Withdrawn dogs	Non-participating/control dogs

### Blood sample collection

Two blood samples from each dog were taken during training season in October 2012 (A samples) and after the race in February 2013 from finishers, withdrawals and controls (B samples). Samples from the three cohorts were further subdivided into A1 and B1 (finishers), A2 and B2 (withdrawals) and A3 and B3 (non-participants/controls) (Table 1).

During the training season, the dogs had rested and fasted for a minimum of 12 h prior to blood sampling. Blood sampling was performed at the mushers’ homes.

Blood samples from the finishers were collected as soon as possible and within 1.5 h after the dogs crossed the finish line. The dogs were not fed prior to blood sampling. Blood samples from the withdrawals were collected at the checkpoints as soon as possible and within 8 h after the dogs were dismissed from the race. Any feeding prior to sampling in these dogs was recorded. Feeding along the trail between the checkpoints was not recorded.

Post-race samples from the controls were collected at the mushers’ homes 2 weeks after the race. The dogs had participated in normal training during the time of the race and the following weeks. They had rested and not been fed for a minimum of 12 h prior to sampling.

### Blood sampling and storage

Blood was retrieved from the cephalic vein; 6 mL in tubes without anticoagulant and 3 mL in EDTA tubes were collected. Tubes without anticoagulant were centrifuged for 10 min after coagulation had occurred and within 1 h after blood collection, and the serum was immediately transferred to empty plastic tubes.

Pre-race samples from all dogs and post-race serum samples from the controls were kept in a cooler. All serum samples were transferred to a freezer (− 70 °C) in approximately 3–4 h, while the EDTA tubes were kept cooled overnight and analysed the next day.

Post-race serum samples from finishers were kept in a cooler and relocated to − 20 °C within 1 h of sampling. The samples from the withdrawals were collected at different checkpoints and kept in a cooler outdoors at approximately − 5 °C after centrifuging. The serum samples from these dogs were transferred to − 20 °C within 2 days after collection. All post-race serum samples were kept frozen during transport and then transferred to − 70 °C for storage at the Norwegian University of Life Sciences. All post-race EDTA tubes were kept cool during the race and analysed the day after the end of the race.

The serum samples were analysed during March 2013.

### Analysis

Analysis of haematological and serum biochemical parameters was performed at The Central Laboratory, Norwegian University of Life Sciences. Serum biochemical parameters, except cortisol, were analysed by ADVIA 1800 (Siemens), and haematological parameters were analysed by ADVIA 2120 (Siemens). Serum cortisol was analysed using IMMULITE 2000 (Siemens). The laboratory’s own reference ranges were employed (Tables 2 and 3).

**Table 2:** Median values and ranges for haematological values in all dogs in training, withdrawals, finishers and controls, along with reference range

Haematology parameters	Reference range	Median pre-race all dogs (n = 46)	Range pre-race	Median finishers (n = 25)	Range finishers	Median withdrawn (n = 9)	Range withdrawn	Median controls (n = 12)	Range controls
WBC	6.0–18.0 × 10e9/L	12.6	7.7–18.4	16.8	8.5–26.6	17.1	9.8–28.4	10.7	7.0–18.1
RBC	5.1–8.5 × 10e12/L	6.98	5.9–7.7	6.21	4.76–7.31	6.74	5.07–7.81	7.85	7.07–8.97
HGB	120–180 g/L	165	133–186	145	111–171	158	114–179	184	172–205
HCT	0.35–0.55 L/L	0.5	0.42–0.56	0.44	0.34–0.53	0.48	0.36–0.55	0.56	0.52–0.63
MCV	62–76 fL	72.4	66.9–77.3	72.6	67.5–76.9	70.6	67.6–79.1	70.9	66.1–76.9
MCHC	320–360 g/L	331	307–346	330	323–338	328	307–377	332	318–350
RDW	11–16%	14.4	12.7–16.7	14.2	12.9–15.4	13.7	12.7–14.9	14	13.2–15.9
PLT	180–500 × 10e9/L	375	201–592	293	202–592	291.5	202–493	303	227–414
NEUT	3.6–13.0 × 10e9/L	7.6	3.6–13.3	13.7	6.8–22.5	14.4	7.6–23.9	6.7	4.0–11.2
LYMPH	0.8–5.8 × 10e9/L	2.5	1.2–3.8	1.6	1–3	1.7	0.8–3.3	2	1.5–4.2
MONO	0–1.6 × 10e9/L	0.6	0.2–1.0	0.8	0.4–1.7	0.8	0.4–2.0	0.4	0.2–0.8
EOS	0–1.8 × 10e9/L	1	0.2–2.1	0.1	0.1–2.1	0.2	0.1–0.7	1	0.3–6.6
BASO	0–0.4 × 10e9/L	0.1	0.0–0.2	0	0.0–0.1	0.1	0.0–0.2	0.1	0.0–0.1
LUC	0–1.5 × 10e9/L	0.2	0.1–0.4	0.1	0.0–0.4	0.1	0.0–0.3	0	0.0–0.1

**Table 3:** Median values and ranges for serum biochemistry values in all dogs in training, withdrawals, finishers and controls, along with reference range

Biochemistry parameters	Reference range	Median pre-race all dogs (n = 65)	Range pre-race	Median finishers (n = 25)	Range finishers	Median withdrawn (n = 21)	Range withdrawn	Median controls (n = 19)	Range controls
AST	0–40 U/L	31	18–92	178	88–758	375.5	89–2539	29	16–156
ALT	0–80 U/L	59.5	35–186	184	94–368	174	62–662	63	31–379
AP	0–90 U/L	37	16–107	61	26–176	79	18–129	34	18–101
CK	0–200 U/L	92	50–558	1294	583–18,918	1344.5	0–67,710	86	39–6209
AMYL	0–1050 U/L	361.5	152–927	274	130–526	204	5–1184	400	213–705
TPROT	54–75 g/L	63	55–71	52	41–62	51	38–64	62	59–68
ALB	32–44 g/L	36	30–44	30	24–37	33	20–45	37	34–44
GLOB	22–31 g/L	27	19–38	21	17–26	21	10–25	24	22–28
A/G	1.0–2.0	1.37	0.79–2.00	1.43	1.13–1.79	1.465	1.04–4.50	1.54	1.26–1.83
UREA	3.5–7.2 mmol/L	6.9	4.0–10.3	14.2	7.1–22.7	13.45	7.6–24.1	6.2	3.5–9.6
CREAT	65–110 µmol/L	76	60–96	56	45–71	53	39–71	70	56–79
BA	0–10 µmol/L	1	0–10	3	1–15	7	1–18	2	0–9
CHOL	3.4–10.0 mmol/L	5.8	4.2–9.1	5.5	4.0–8.1	5.6	3.4–8.9	5.2	3.4–7.9
GLU	3.6–6.6 mmol/L	4.8	3.5–6.4	5.5	4.0–7.6	7.5	3.9–12.6	5.9	4.8–6.9
PHOSP	0.9–2.0 mmol/L	1.2	0.7–1.6	1.4	0.9–1.9	1.6	1.0–2.3	1.2	0.8–1.8
Ca	2.2–2.9 mmol/L	2.5	2.2–2.7	2.2	2.0–2.4	2.15	1.9–2.4	2.4	2.3–2.5
Na	140–154 mmol/L	148	146–151	148	146–152	147	142–151	149	147–151
K	3.7–5.8 mmol/L	4.7	3.9–5.3	3.8	3.3–4.3	3.85	3.1–4.7	5.1	4.8–5.5
Na/K	> 27.0	31.3	27.9–37.7	39.5	34.4–45.8	37.9	30.6–46.8	29.4	26.7–31.5
Cl	99–115 mmol/L	113	107–118	116	111–123	113	108–120	113	110–116
CRP	0–15 mg/L	18.7	1.2–78.9	75.1	27–192	93.05	22–220	3.2	0.0–156.9
CORT	20–250 nmol/L	58	14–141	147	74–323	80.5	46–224	52	34–125

### Statistical analysis

**Fig. 1:**
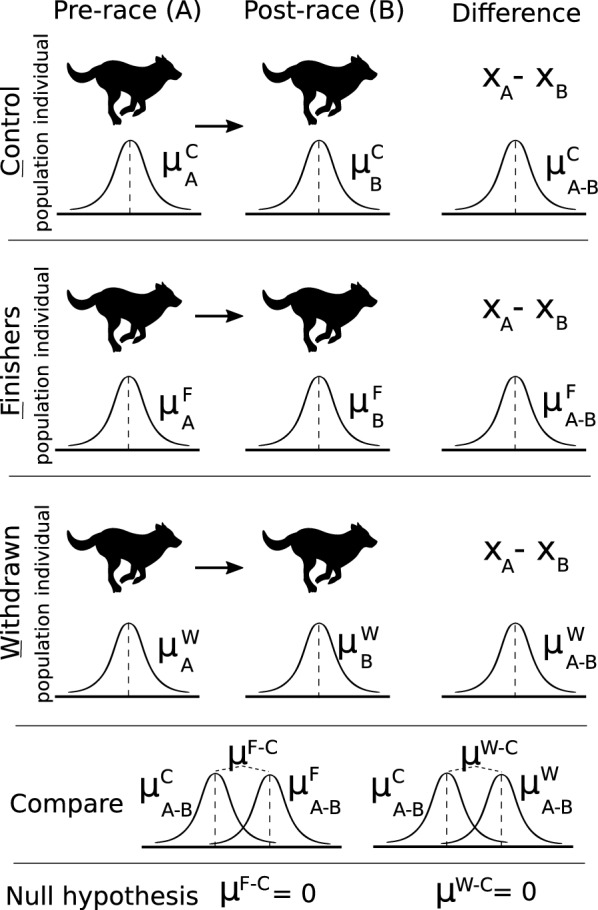
Study design and the comparisons between the cohorts; withdrawals, finishers and controls

All statistical analyses were performed in R 3.3.1 [8]. Variable correlation was explored using correlation plots from the *psych* package [9]. The variable breed was excluded from further analysis due to high collinearity to owner. Feeding regimen was left out due to a lack of variation. In the following, biochemistry and haematological variables were converted to represent the (absolute) change in the variable between post-race (B samples) and pre-race (A samples). Our statistical model thus focused on (average) changes within a single dog. First, all A-samples were compared by ANOVA (or, in the case of severely non-normally distributed parameters such as creatine kinase, Kruskal–Wallis tests) to evaluate homogeneity between the cohorts prior to racing. Cohorts were found to be comparable for all parameters (data not shown). Associations with demographic variables were carried out using 1000 bootstrap replicates of a linear regression model with the following variables used as predictors: Cohort (with control dogs as the reference group), owner, sex (reference: female) and age (< or ≥ 5 years). For each bootstrap replicate, the covariate coefficients were kept. The median coefficient was then extracted from the list of 1000 bootstrap replicates. The P-value was calculated as followed; First, approximate normality of the bootstrapped coefficient list was asserted through normality plots (not shown). Then, a Student’s t-value was calculated by dividing the mean of the coefficient list by the standard deviation of the list. We then used the distribution function for Student’s t (with the degrees of freedom left in the model) to get the P-value for this result. (Null hypothesis: that this list of 1000 coefficient covariates could have been sampled if the true coefficient value was 0, i.e., that the coefficient is significantly different from 0). The parameter creatine kinase (CK) deviated significantly from normality but conformed well to a normal distribution after taking the logarithm. The parameters lipase and total bilirubin could not be transformed to normality and were later excluded from analysis. For these parameters, we evaluated difference in cohorts with the Kruskal–Wallis test instead. In the boxplots of Fig. 1 we first used ANOVA (on the logarithmic transformations) to investigate any cohort differences, then t-tests to check for any between-group differences. For all of the above analyses, we used a significance threshold of < 0.0001 due to the high number of comparisons being undertaken. In addition, demographic covariate interactions were explored but were not included in our final models due to low model support, as evaluated by likelihood-ratio tests between the simpler and interaction-term models.

Study design and the comparisons between the different cohorts are illustrated in Fig. 1.

## Results

Three kennels with a total of 47 Alaskan huskies and one kennel containing 18 Siberian huskies were included. There were 32 females and 33 males with a mean age of 3.8 years (range 1–9 years). Forty-six dogs were race participants, while 19 dogs served as controls. Three mushers and 25 dogs completed the race within 3 to 4 days. Twenty-one dogs, including a complete team of 12 dogs, were dismissed at different times during the race.

Each dog was considered healthy at the time of pre-race as well as at post-race sampling when referring to the controls and finishers, as no clinical signs of illness were observed. Withdrawals were dismissed due to lameness, depression, anorexia and/or dehydration. The main reason for withdrawal of a whole team was anorexia in several dogs. Seven withdrawals had been fed at the checkpoint prior to blood sampling. Based on information from the mushers, collected via questionnaires, training and feeding routines prior to the race were considered equal for all dogs.

Due to storage failure, 19 pre-race EDTA samples had to be excluded, leading to removal of the matching post-race samples. Hence, the number of EDTA samples included was 9 in the withdrawal group and 12 in the control group.

The median and range values from the three cohorts before training and post-race are presented in Tables 2 and 3. Results for serum biochemistry and haematological values in the regression model, where the variables age, sex and owner are included, are listed in Additional file 1.

### Haematology

The increase in neutrophil cell count was significantly greater in withdrawn dogs (P < 0.0001), and the decrease in erythrocytes (RBC), haematocrit (HCT), haemoglobin (HGB) and eosinophils was significantly lower (P < 0.0001) compared to the controls.

In finishers, the increase in white blood cell count (WBC), large unstained cell count (LUC), neutrophils and monocytes was significantly greater (P < 0.0001) compared to controls, while the decrease in RBC, HCT, HGB and eosinophils was significantly lower (P < 0.0001).

RBC, HGB and HCT were significantly higher and LUC significantly lower in the controls’ B samples (P < 0.0001) compared to results from the A samples from all three cohorts during training.

No haematological parameters showed any significant difference in owner, gender or age effect.

### Serum enzyme activities

**Fig. 2:**
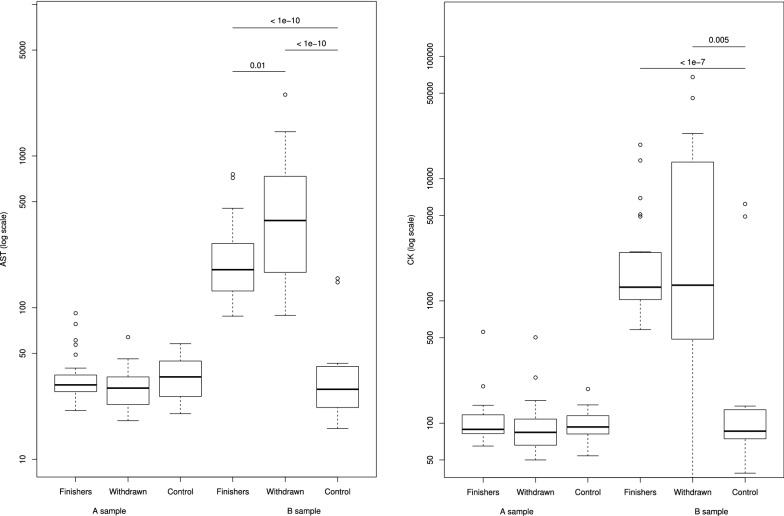
Box plot with logarithmic presentation for aspartate aminotransferase values and creatine kinase values by the subgroups finishers (n = 25), withdrawals (n = 21) and controls (n = 19). Statistically significant between-group differences are highlighted with P-values from the t-test

The increase in aspartate aminotransferase (AST), CK (log scale) (Fig. 2), alanine aminotransferase (ALT) and alkaline phosphatase (AP) were significantly greater in withdrawals only (P < 0.0001) compared to controls.

### Blood urea nitrogen and creatinine concentrations

The increase in blood urea nitrogen (BUN) was significantly higher and the decrease in creatinine concentration significantly lower in both withdrawals and finishers compared to controls (P < 0.0001). Additionally, the decrease in creatinine was significantly lower in the B-samples from the controls compared to A-samples from all three cohorts (P < 0.0001).

### Serum protein concentrations

The decrease in albumin, globulin and total protein was significantly lower in both withdrawn and finishing dogs compared to controls (P < 0.0001). Albumin and globulin were significantly influenced by the owner variable (P < 0.0001).

### Glucose and cholesterol

The increase in glucose was significantly higher and the decrease in cholesterol significantly lower in B samples from the controls compared to A samples from all three cohorts (P < 0.0001).

### Electrolytes and mineral concentrations

In withdrawals, the elevation in phosphorus concentration was significantly higher compared to controls (P < 0.0001). The decrease in calcium and potassium levels were significantly lower (P < 0.0001) and the increase in sodium/potassium significantly higher in both withdrawals and finishers (P < 0.0001). The decrease in calcium was significantly lower in the controls when comparing their B samples to all A samples from the three cohorts (P < 0.0001). The level of phosphorus was significantly influenced by the owner (P < 0.0001).

### C-reactive protein and cortisol

The elevation in C-reactive protein (CRP) was significantly higher in both withdrawals and finishers compared to controls (P < 0.0001), and the increase in cortisol was significantly greater only in the finishers (P < 0.0001).

No biochemistry parameters were influenced by age or gender.

**Fig. 3:**
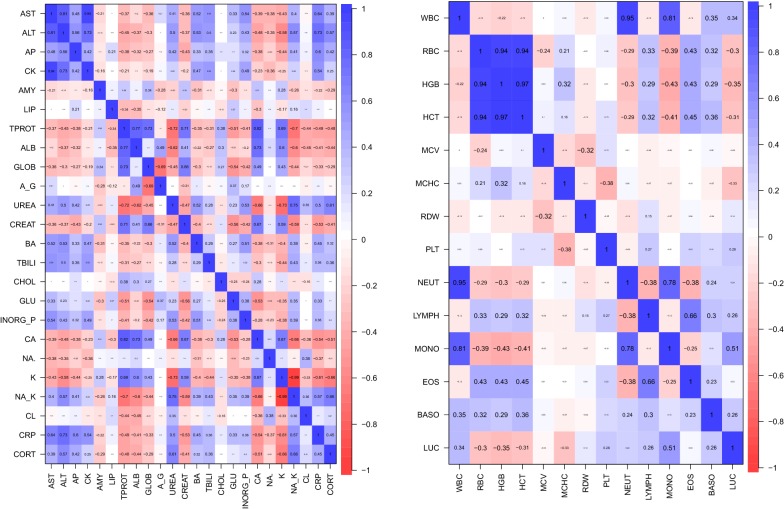
Correlation plots illustrating positive (blue colour) and negative (red colour) correlations between the different haematological and serum biochemistry parameters

Correlation plots for haematology and biochemistry parameters are presented in Fig. 3.

## Discussion

The number of scientific publications presenting complete haematological and serum biochemistry profiles in dogs participating in endurance activities is limited. This report includes a survey of both haematological and biochemical parameters from the same individuals before and after long distance racing. Stress prior to a race may influence the results of blood analysis [10]. By comparing post-race results with outcomes from samples taken during the training season, and not immediately prior to race start, the possible impact from anticipation, stress and environmental challenges normally present at this stage, was eliminated.

Many significant findings observed in this study were found both in the withdrawals and the finishers, most of them were minor and relative changes within reference ranges. Studies regarding estimates of biologic variation have revealed high individual variation of many routinely measured analytes [11, 12]. Hence, biologic variation may have accounted for minor alterations in the blood parameters analysed. This might indicate that many of the observed changes simply reflect physiological adaption due to endurance exercise, and that the majority of racing sled dogs cope well.

However, significant increases in the muscle enzymes AST and CK were seen in the withdrawn group when compared to the control dogs. Unlike most other significant findings, median values of muscle enzymes were also well above reference range in this group. Some of the withdrawals, demonstrating particularly elevated muscle enzymes, had clinical signs that could be related to rhabdomyolysis. Other studies also report that elevations in muscle enzymes in racing sled dogs can be clinically relevant, and might indicate rhabdomyolysis and performance failure [13–15].

Moderate increases in AST and CK due to musculoskeletal catabolism are also expected in healthy sled dogs [1, 2, 13, 14, 16]. The median values of CK and AST were also above the upper reference range in the finisher group. However, significant changes were not detected when comparing the finishing cohort to the control group. C-reactive protein was significantly increased in both withdrawn and finishers. In addition, CRP was above reference range, which may reflect an interaction with muscle degradation and an inflammatory response, also stated previously [17].

The significant elevations in ALT and AP among the withdrawals may reflect a reactive hepatopathy, possibly secondary to increased metabolic demands and gastrointestinal dysfunction during heavy exercise. Similar changes are observed in marathon runners, post-exercise [18]. It has also been suggested that an increase in ALT in racing sled dogs is due to muscle degradation, rather than hepatic damage [2, 19].

There has been speculation whether racing sled dogs may experience transient kidney dysfunction [20], which has been reported in human marathon runners [21]. In this study, both withdrawals and finishers showed a significant decrease in creatinine concentration and a significant increase in BUN. However, these findings were relative changes within reference range. This correlates with most previous reports [1, 13, 14]. A relative increase in BUN with normal or decreased creatinine is thought to be the result of high dietary protein intake combined with increased protein catabolism during racing [2, 14].

Significant decreases in RBC, HGB and HCT, as well as globulin, albumin and total protein, were observed in both withdrawals and finishers when compared to controls. Still, all findings were relative changes within reference range. These results correlates with previous reports [3, 14], and are mainly thought to be caused by plasma volume expansion during physical exercise [1–3, 14, 22]. However, sled dogs during endurance racing are at high risk of developing gastrointestinal illness, like enteritis, gastritis and gastric ulceration [5], and gastrointestinal loss of red blood cells and proteins cannot be excluded as cause of the observed changes in HCT and proteins, both in withdrawals and finishers [6, 23].

Both withdrawn and finishing dogs experienced a significant increase in neutrophils, as well as a decrease in eosinophils compared to controls. In finishers, significant increases in WBC and monocytes were also observed. A similar WBC response is observed during long-distance running, both in humans and sled dogs [3, 24], which is an expected physiological response during heavy exercise [3]. An increase in endogen cortisol is considered partially responsible for the observed stress leukogram observed after exercise in humans and horses [25, 26], and should also be suspected as a cause in canine athletes. The significant elevation in cortisol observed in the finishing dogs in this study, supports the exercise-induced increase in cortisol recognized in sled dogs in previous studies [27, 28].

### Limitations

It was not possible to completely standardize storage of the blood sample. Time from sampling to analysis differed between some of the groups and may have affected especially the haematological results [29]. Delayed sampling in some of the withdrawals may also have allowed for some degree of restoration, and the observed changes may have been underestimated, especially in parameters with short serum half-life. Some withdrawals were fed prior to post-race sampling. This may have had an impact on some of the blood results, however, CK and AST results should not be markedly influenced.

The Central laboratory is a reference laboratory with high level of quality assurance, however, all biochemical samples could not be run the same day which may have increased analytical imprecision.

Some haematological samples were unfortunately not included in the statistical analysis due to storage failure of their pre-race EDTA tubes. However, the number of remaining samples was still sufficient to support the statistical significance in the presented results.

## Conclusions

The majority of the statistically significant biochemical and haematological changes in dogs competing in a 600 km race were minor and often within reference range. Although often more pronounced in withdrawn dogs, significant changes were also observed in the finishing group, probably reflecting physiological adaption due to exercise rather than illness. However, only withdrawals experienced a significant elevation in muscle enzymes with values high above reference range, indicating muscle damage as a possible explanation for being retired from the race.

## Additional file

**Additional file 1.** Coefficients from the multivariable regression analysis, explaining how each demographic variable (cohort, age, sex and owner) relates to a particular haematological/serum biochemistry parameter.

## Abbreviations

CK: creatine kinase
RBC: erythrocytes/red blood cell count
HCT: haematocrit
HGB: haemoglobin
WBC: white blood cell count
LUC: large unstained cell count
AST: aspartate aminotransferase
ALT: alanine aminotransferase
AP: alkaline phosphatase
BUN: blood urea nitrogen
CRP: C-reactive protein
