# Supplementary material for: Haematological and serum biochemical values in Norwegian sled dogs before and after competing in a 600 km race
Source: Acta Vet Scand. 2019 Apr 25;61:20. doi: 10.1186/s13028-019-0453-5 (PMC6485113; doi:10.1186/s13028-019-0453-5)
Supplement: Supplementary file 1 — Additional file 1. Coefficients from the multivariable regression analysis, explaining how each demographic variable (cohort, age, sex and owner) relates to a particular haematological/serum biochemistry parameter. [file 13028_2019_453_MOESM1_ESM.pdf]

Additional file 1 (pdf)

Additional file 1. Coefficients from the multivariable regression analysis, explaining how each demographic variable (cohort, age, sex and owner) relates to a particular haematological/serum biochemistry parameter.

	Intercept		Cohort (Reference group = control)			
	B - A		Finishers		Withdrawals	
	Estimate	p	Estimate	p	Estimate	p
**Aspartate aminotransferase**	-94	0.203	45.7	0.678	549	**< 0.0001**
**Alanine aminotransferase**	-52.4	0.074	70	0.06	171.3	**< 0.0001**
**Alkaline phosphatase**	-13.9	0.09	39.6	0.002	43.5	**< 0.0001**
**log(Creatine kinase)**	2.6	0.008	2.9	0.006	4.9	**< 0.0001**
**Amylase activity**	42.7	0.313	-90.1	0.036	-159.9	0.001
**Total protein**	-3.1	0.048	-11.2	**< 0.0001**	-10.4	**< 0.0001**
**Albumin**	0.2	0.875	-7.2	**< 0.0001**	-6.3	**< 0.0001**
**Globulin**	-3.5	0.004	-4	**< 0.0001**	-4.1	**< 0.0001**
**Albumin-globulin ratio**	0.4	0.051	0	0.904	0.1	0.455
**Blood urea nitrogen**	0.4	0.553	9.1	**< 0.0001**	7.6	**< 0.0001**
**Creatinine**	-14.7	**< 0.0001**	-16.1	**< 0.0001**	-17	**< 0.0001**
**Bile acids**	1.7	0.205	1.9	0.091	4	0.002
**Cholesterol**	-1.4	**< 0.0001**	0.6	0.114	1.1	0.003
**Glucose**	1.9	**< 0.0001**	-0.1	0.784	0.8	0.065
**Inorganic phosphate**	-0.1	0.199	0.1	0.184	0.4	**< 0.0001**
**Calcium**	-0.2	**< 0.0001**	-0.2	**< 0.0001**	-0.2	**< 0.0001**
**Sodium**	1.5	0.014	0	0.952	-1.8	0.011
**Potassium**	0.4	0.002	-1.5	**< 0.0001**	-1.1	**< 0.0001**
**Sodium-potassium ratio**	-2.1	0.039	11.6	**< 0.0001**	8	**< 0.0001**
**Chloride**	-0.5	0.542	2.5	0.001	0.7	0.35
**C- reactive protein**	-23.4	0.046	68.2	**< 0.0001**	105.6	**< 0.0001**
**Cortisol**	-20	0.086	88.5	**< 0.0001**	47.2	0.001

	Owner (Reference group = 1)					
	2		3		4	
	Estimate	p	Estimate	p	Estimate	p
**Aspartate aminotransferase**	161	0.142	454	0.072	261.7	0.042
**Alanine aminotransferase**	52.3	0.173	136.2	0.048	94.6	0.025
**Alkaline phosphatase**	21.1	0.052	11.1	0.53	7.7	0.524
**log(Creatine kinase)**	1.4	0.094	2.3	0.004	1	0.196
**Amylase activity**	-25.7	0.566	-46.7	0.413	-103.9	0.082
**Total protein**	5.6	0.004	5.6	0.014	0.9	0.617
**Albumin**	6.5	**< 0.0001**	0.6	0.769	-1	0.535
**Globulin**	-0.8	0.527	5.1	**< 0.0001**	2	0.064
**Albumin-globulin ratio**	0.1	0.525	-0.4	0.047	-0.3	0.1
**Blood urea nitrogen**	-4.1	0.001	-3.2	0.037	-0.3	0.819
**Creatinine**	7.2	0.041	12.5	0.001	11	0.001
**Bile acids**	-2.4	0.056	0.4	0.799	-0.5	0.712
**Cholesterol**	0.6	0.162	0.4	0.347	-0.2	0.504
**Glucose**	-1.3	0.016	-1.2	0.024	-0.4	0.406

**Inorganic phosphate**	0.1	0.478	0.3	**< 0.0001**	0.3	0.005
**Calcium**	0.2	0.001	0.1	0.044	0	0.75
**Sodium**	-0.2	0.839	-1.3	0.2	-1.3	0.174
**Potassium**	0.1	0.3	-0.1	0.487	-0.2	0.348
**Sodium-potassium ratio**	-2	0.067	-0.4	0.827	1	0.528
**Chloride**	1.2	0.164	1.3	0.293	1.7	0.055
**C- reactive protein**	18	0.314	28.7	0.254	13.6	0.4
**Cortisol**	-1.4	0.913	45.3	0.086	12.1	0.452

	**Gender (Reference group = F)**		**Age (Reference group ≤ than 5)**	
	**Male**		**Age**	
	**Estimate**	**p**	**Estimate**	**p**
**Aspartate aminotransferase**	19.9	0.868	-206.2	0.005
**Alanine aminotransferase**	44.8	0.116	-44.2	0.045
**Alkaline phosphatase**	1	0.898	-8.3	0.335
**log(Creatine kinase)**	0.7	0.165	-0.8	0.098
**Amylase activity**	-24.7	0.408	83.3	0.048
**Total protein**	0.1	0.903	-1	0.539
**Albumin**	0.2	0.863	-1.6	0.321
**Globulin**	0	0.974	0.5	0.564
**Albumin-globulin ratio**	-0.1	0.422	-0.2	0.153
**Blood urea nitrogen**	0.6	0.482	0	0.949
**Creatinine**	2.2	0.248	1.2	0.622
**Bile acids**	1	0.321	-1.2	0.293
**Cholesterol**	0.1	0.729	-0.2	0.511
**Glucose**	-0.2	0.588	-0.3	0.433
**Inorganic phosphate**	0.1	0.316	0	0.345
**Calcium**	0	0.906	0.1	0.171
**Sodium**	-0.7	0.177	0.3	0.586
**Potassium**	0	0.973	-0.1	0.244
**Sodium-potassium ratio**	0.4	0.625	1.1	0.182
**Chloride**	-1.1	0.089	0	0.965
**C- reactive protein**	7	0.525	-16.5	0.227
**Cortisol**	3.8	0.809	19.6	0.259

	**Intercept**		**Cohort (Reference group = control)**			
	**B - A**		**Finishers**		**Withdrawals**	
	**Estimate**	**p**	**Estimate**	**p**	**Estimate**	**p**
**White blood cell count**	-1.4	0.281	6.2	**< 0.0001**	7.3	0.001
**Red blood cell count/erythrocytes**	1.3	**< 0.0001**	-2.1	**< 0.0001**	-1.5	**< 0.0001**
**Haemoglobin**	32	**< 0.0001**	-52.2	**< 0.0001**	-37.2	**< 0.0001**
**Haematocrit**	0.1	**< 0.0001**	-0.1	**< 0.0001**	-0.1	**< 0.0001**
**Mean cell volume**	-0.2	0.757	0.6	0.312	1.6	0.161
**Mean corpuscular haemoglobin concentration**	4.6	0.322	-7	0.139	-8.2	0.174
**Red cell distribution width**	-0.5	0.229	0.4	0.219	0	0.895
**Platelet cell count**	-89.6	0.013	21.7	0.45	30.3	0.443
**Neutrophil cell count**	-1.1	0.355	6.9	**< 0.0001**	9.1	**< 0.0001**

**Lymphocyte cell count**	-0.5	0.008	-0.4	0.029	-0.9	0.004
**Monocyte cell count**	-0.1	0.366	0.4	**< 0.0001**	0.3	0.02
**Eosinophil cell count**	0.2	0.125	-0.9	**< 0.0001**	-1.2	**< 0.0001**
**Basophil cell count**	0	0.917	0	0.065	0	0.353
**Large unstained cell count**	-0.1	**< 0.0001**	0.1	**< 0.0001**	0.1	0.051

	**Gender (Reference group = F**		**Age (Reference group ≤ than 5)**	
	**Male**		**Age**	
	**Estimate**	**p**	**Estimate**	**p**
**White blood cell count**	-0.4	0.759	-0.8	0.528
**Red blood cell count/erythrocytes**	0.3	0.149	-0.6	0.006
**Haemoglobin**	8	0.128	-11.7	0.077
**Haematocrit**	0	0.222	0	0.042
**Mean cell volume**	-0.6	0.315	1.7	0.013
**Mean corpuscular haemoglobin concentration**	6.4	0.085	-2.5	0.585
**Red cell distribution width**	-0.3	0.287	0.3	0.224
**Platelet cell count**	19	0.39	14.4	0.487
**Neutrophil cell count**	-0.1	0.936	-0.9	0.455
**Lymphocyte cell count**	0	0.952	0.2	0.322
**Monocyte cell count**	-0.1	0.247	-0.1	0.541
**Eosinophil cell count**	-0.2	0.209	-0.1	0.443
**Basophil cell count**	0	0.771	0	0.304
**Large unstained cell count**	0	0.502	0	0.637
